# The Time in Which We Think

**Published:** 1888-09

**Authors:** 


### The Time in Which We Think.

One of the most beautiful applications of electricity which has of late been made is its use in the study of psychological phenomena. And why, indeed, is not the subtle power by which time and space are being annihilated, and human labor rendered less irksome, the most proper agent to assist man in the study of the facts of his own consciousness? In an elaborate article in the *Nineteenth Century*, Dr. J. McK. Cattell, gives an account of the time-measurements of thought made by means of the line drawn on a rapidly moving surface by a pen attached to the prong of a tuning fork vibrating at a constant rate, by means of electricity. By a delicate apparatus constructed on this principle, duration of time may be measured to the one ten-thousandth of a second. The writer above named has found that the process of thought varies in its degree of rapidity in different individuals, children and old persons thinking slower than people of middle age, ignorant persons thinking more slowly than educated persons. In this way he also found that he could measure the time it takes to perceive, that is, the time which passes from the moment when the impression reaches consciousness until the moment at which we know what it is. In his own case he found that it took 1–20 second to see white light, 1–10 second to see a picture, 1–8 to see a letter, and 1–7 to see a word. It takes a longer time to see a rare word than a common word, or a word in a foreign language than in our native tongue. It even takes longer to see some letters than others. "Will time," or time taken up in choosing can be measured. It takes 1–13 seconds to judge between blue and red. To recall the name of a printed word takes 1–9 second, to a letter 1–6 second, to a picture 1–4 second. It takes less time to remember the name of a familiar word than of a letter, though it takes less time to see the letter. The time of remembering can be measured. It takes 1–4 second to translate a word from one language to another when one is familiar with both. It takes 1–20 second longer to translate a word from a foreign language to one's native tongue than it does in the other direction. We can think of the name of the next month in half the time we can think of the last month. It has been demonstrated that sensation does not travel through the nerves to the brain so fast as has been supposed. Its speed is not much greater than sixty miles an hour.—*Light and Heat.*

---

Medical Society on Japan.—In the report of the proceedings of a special meeting of the Sei I Kwai or Medical Society of Japan, held at Tokyo, on November 30, 1887, *The Australasian Medical Gazette* says: The first business which occurred was the introduction by the President, Takaki-Kanehiro, F.R.C.S., Eng., of Miss Light, M.D., a recently-elected member of the Society, practicing in Tokyo. This election shows a liberal and enlightened spirit we cannot too highly eulogize. Many of our readers may not be aware of the prominent position which is taken by modern European medicine in Japan, we therefore think it not out of place to say that the leading practitioners, who are really numerous, possess diplomas of the highest character, which they obtained after study in Europe, and the papers read at and the reports of the meeting of this society show how highly cultivated these gentlemen are, and how enthusiastically and thoroughly, medicine, surgery, and sanitation are practiced in Japan, which but thirty years since was a sealed country against all European knowledge. The action of the State in these matters is far in advance of that of the Australian Government, and the reports of the medical department of the Japanese Imperial Navy are almost a lesson to the world.

An Old Idea Patented.—Dr. Joel W. Smith, Charles City, Iowa, writes: "The Farny Suture," said to be patented July 13, 1886, in England, France, Austria, etc., consists in the application of adhesive plaster, upon each side of a wound, (wherever skin sutures or adhesive straps have usually been made use of to bring the divided or weakened parts in close apposition, and so as to favor the healing process) with eyelets at the end or side of each plaster nearest the wound, and thus permitting the lacing of two plasters together.

The writer can testify that it is a valuable substitute in many cases for long plaster strips or skin sutures, after having repeatedly made use of the same thing, and in the earlier years of professional life, beginning 1850. The patent of course, could not be sustained, but it is well to have it widely advertised.

Mucous Patches.—A solution of chromic acid is perhaps the best application to mucous patches, especially to those in the mouth and the pharynx. Use from two to five grains to the ounce.

Dr. W. B. Hills, of Cambridge, read a paper on "The Value of Corrosive Sublimate as a Practical Disinfectant." The author said that disinfection is now based on the presence of micro-organisms, and hence disinfectants are only those substances which will destroy the vitality of the micro-organisms. The hypochlorites are efficient on account of their oxidizing power, and this destroys other organic substances as well as the germs. Previous to 1880 corrosive sublimate had not been shown experimentally to possess the power. Koch says 1:5,000 aqueous solution will kill almost all germs, and 1:10,000 will destroy all of them. 1:1,000,000 restrains partially the growth of spores of the anthrax bacillus, and 1:300,000 absolutely restrains them. Corrosive sublimate is now at the head of the list of the disinfectants recommended by the Committee on Public Health. Its power, however, varies with the substances acted upon, being greatest for aqueous solutions and least for albuminous, since there is found an insoluble albuminate of mercury. It is true that some of the germs must be confined between the pacticles of this, but they are not destroyed and may become free again. Hence, for disinfection, corrosive sublimate must be used in excess, *i. e.*, allowance must be made for the chemical change. It is, moreover, destructive to lead pipes, and hence should not be disposed of through them. It may be used to wash woodwork, furniture, etc., and clothes not stained with excreta (which are albuminous), but it is absurd to use it for large quantities, *e. g.*, vaults, cesspools, etc. The excreta should be disinfected before going into the vaults. The danger of poisoning is very slight; for the solutions are so dilute and the taste is bad. It is, however, risky if not dangerous to use so much in sprinkling the streets, which may rise again as dust.

The size of drops, according to Mr. A. F. Reid, (*Chem. News*) other conditions being the same varies with the rapidity of the dropping. A pipette which delivered 136 drops, at the rate of one in two seconds, delivered 141 at the rate of two in a second. With increase of temperature, also, there is a notable decrease in the size of drops, even in the case of mobile fluids like water.

